# Integration of Palliative Care Into All Serious Illness Care as A Human Right

**DOI:** 10.1001/jamahealthforum.2021.1099

**Published:** 2021-04-01

**Authors:** William E. Rosa, Betty R. Ferrell, Diana J. Mason

**Affiliations:** Department of Psychiatry and Behavioral Sciences, Memorial Sloan Kettering Cancer Center, New York, New York; City of Hope National Medical Center, Duarte, California; Center for Health Policy and Media Engagement, George Washington University School of Nursing, Washington, DC

Deaths from COVID-19 are approaching 600 000 in the US and 3 million worldwide. As intensive care units have reached or exceeded capacity in many hospitals, concerns have grown about the ethical dimensions of rationed care. But many of these focused on who gets a ventilator, rather than how to provide palliative care to those who are seriously ill, including those treated with ventilators and those who may not want or cannot get a ventilator.

Early on in the pandemic, clinical leaders at Mount Sinai Medical Center in New York City, New York, realized that although they had a palliative care team, they did not have enough clinicians prepared to lead the crucial conversations with patients and families about end-of-life decision-making and symptom management.^1^ There is always a consistent need for the deep expertise of palliative care teams, but COVID-19 has highlighted that every clinician needs knowledge and skills in the fundamentals of palliative care.

Focusing primarily on access to ventilators sets up a mental model that fails to capture the premise of person-centered care. This model frames the rationing decisions in terms of abandoning the patient if the health system and clinicians cannot provide a ventilator. But patient abandonment only occurs if decision-making and care stop at the point of the ventilator. Palliative care is not dependent on life-saving interventions. It may very well include a ventilator, but it is ultimately structured around the individualized wants and needs of the patient. It includes alleviating suffering and managing complex communications, psychosocial dynamics, fluctuating symptom management needs, and spiritual care throughout the dying process.

Every patient treated with a ventilator also needs palliative care. It is not an either-or clinical proposition, but rather a both-and moral imperative.

COVID-19 has accentuated the need for clinicians to have frequent conversations with patients and families about dying. The pandemic has forced many healthy people to confront rapid-onset, life-threatening trajectories of acute illness. Patients are dying without their loved ones, and families are grieving alone. For every person who dies, an average of 9 others are profoundly affected and grieve.^2^ COVID-19 has interrupted the cultural and community practices for coping with death, raising concerns about the pervasiveness of grief and loss associated with the pandemic. This disruption is reflected in the US Centers for Disease Control and Prevention’s guidance on coping with grief and loss during the pandemic. Frontline generalist clinicians trained in the fundamentals of palliative care are essential for ensuring appropriate support for families’ anticipatory grief prior to a loved one’s death, and extending into the bereavement phase, for sharing resources, for coping with loss, and for identifying when complicated grief requires a referral for care.

The mandate for universal access to palliative care preceded COVID-19. In 2014, the World Health Assembly called for the fundamentals of palliative care to be embedded in the care provided by health care systems and integrated into all clinicians’ training. The US population is aging, and structural barriers to care (such as systemic racism and disparities in insurance coverage and access to care) increase the burdens of chronic illness. The need for palliative care for those dying from serious illness is expected to increase approximately 87% over the next 4 decades, disproportionately affecting people living in poverty and people older than 70 years, among other groups. Although 72% of US hospitals with 50 beds or more report having a palliative care team, 90% of them are in urban areas, rendering palliative care services out of reach for many in rural communities. In addition to palliative care in hospitals, enhanced efforts are needed to provide people with community-based alternatives for care that use a palliative care framework.

Clinicians are suffering, too.^3^ We are well aware of the long history of clinician burnout among hospital physicians and nurses that has escalated during the COVID-19 pandemic. There have been calls for tracking the psychological well-being of clinicians, but this is not sufficient to address the moral injury that so many are experiencing. Training clinicians with generalist palliative care skills may decrease their burnout and moral distress by providing them with the ability to navigate clinical uncertainty with a greater sense of confidence. At 6 months after a primary palliative care communication skills training program, oncology advanced practice registered nurses reported increased participation in family meetings, more referrals to palliative specialists, more family conversations about bereavement resources, and more active preparation of clinical staff for patient deaths.^4^

Medical and nursing schools can play a pivotal role in preparing clinicians with these critical skills. To date, the End-of-Life Nursing Education Consortium, in partnership with the American Association of Colleges of Nursing, has integrated palliative care education into 570 undergraduate and 210 graduate programs across the US. More than 40 outcome studies on these programs have been published in the US and globally.

The Palliative Care and Hospice Education and Training Act, which passed the US House of Representatives in 2019, would require that the US Department of Health and Human Services establish palliative care education centers and encourage other actions to increase access to palliative care and increase public awareness of its benefits. Although introduced in the US Senate in 2019, it made no headway there during the congressional term. With mounting deaths and grief, it is time to try again for its passage.

Also needed are payment models that prioritize clinician-patient relationships and high-burden needs of patients with serious illness while reducing administrative stress and linking fiscal reward to improved outcomes. Capitated approaches are more likely to encourage clinicians to focus on what patients need and want. Yet outcomes-based payment approaches are complicated, because death is never considered an acceptable outcome and paying for family-centered care is still not part of most discussions of payment reform. Furthermore, existing payment models address palliative care as a specialty, not a generalist function.

Palliative care as a philosophy and a clinical specialty is an indispensable approach to narrowing disparities and promoting equitable health services through person-centered, family-centered, and community-centered engagement.^5^ Historically marginalized communities are in urgent need of palliative care interventions and expertise during the pandemic. Black, Latinx, Native American and Indigenous, and other people of color have been disproportionately affected by COVID-19 in terms of both mental health consequences and severe illness. Palliative care health equity frameworks promote assessment of the social and moral determinants of health, as well as systemic injustices, to tailor services to the individual, context, and culture at hand.^6,7^ Other marginalized populations, such as lesbian, gay, bisexual, transgender, and other sexual and gender minority persons and their families of choice, require greater attention to inclusive palliative care practices during COVID-19 that foster trust, transparency, and value-concordant care delivery.^8^

Access to palliative care is a human right. Our inability to deliver it in the setting of COVID-19 and other serious illnesses is a human rights violation. Each of us is a stakeholder. Health systems and clinicians are charged with meeting the holistic needs of patients and family caregivers in the face of serious illness. Education, payment, and health equity reforms are needed now to hold clinicians accountable to those we serve.
